# Gallic Acid in Hemorrhage from the Urinary Organs

**Published:** 1884-08

**Authors:** Lionel S. Beale


GALLIC ACID IN HEMORRHAGE FROM THE URINARY ORGANS.

Of the styptics in ordinary use, gallic acid, according to my experience, is one of the most potent in relieving hemorrhage from the urinary organs. The reputation of this remedy would, I think, soon be greater than it is if those who try it would give it in sufficiently large doses, and persevere in it use for several days before replacing it by other astringents. As gallic acid probably acts according to the strength of its solution which bathes the bleeding tissue, it is necessary to insure the introduction of a certain quantity into the blood by the frequent administration of successive doses. We must remember that gallic acid soon passes away from the blood, being carried off in the urine. It is, therefore, only by administering frequent doses that we can hope to compensate for this continual draining away of the remedy, and we must give it in quantity and often enough to more than compensate for what is removed with excrementitious matters.

In chronic bleeding from the surface of the mucous membrane of the pelvis of kidney, ureters, bladder and urethra, and from villous growths, as well as in the very obstinate hemorrhage from large fungous tumors of the kidney and bladder, I have found gallic acid most valuable in a large number of cases, and for some years past I have been led to depend upon it more and more. In that spongy condition of the prostate, when the veins are large and the capillaries of the surface considerably dilated and forming here and there little pouches like aneurismal dilatations, hemorrhage is often not only very obstinate, but, from time to time, in such excessive quantity as to blanch and weaken the patient. The remedy should be give in frequent doses, day and night, until the bleeding is very decidedly reduced in degree, when it may be ordered once in six hours, or less frequently, being again increased in frequency, if the patient ceases to improve, or the hemorrhage again increases in severity.

Gallic acid seldom disagrees in any way. Some patients complain of its taste, but it is generally well borne by the stomach. It does not cause constipation, and even when the crystals are swallowed in a state of suspension in water or mucilage, no inconvenience results, and the stomach is not disturbed by their presence. The glycerine of gallic acid is, however, the most pleasant form in which to prescribe the remedy. This contains one part of gallic acid in four. Forty minims will contain ten grains, and may be given in distilled water, peppermint, orange or other water. But it is most essential that the patient should persist in taking the doses regularly for several days. Gallic acid is absorbed by the blood, and passes away unchanged in the urine; and it is probable that it acts directly on the parts from which the bleeding is taking place, and therefore a certain strength of solution is necessary to get the good effects, and this can only be obtained by its persistent introduction into the stomach, and so into the blood, at short intervals of time. I have given gallic acid in ten-grain doses every three hours without intermission for three weeks, no objection having been made on the patient's part. Whether much larger doses would be absorbed I doubt, but I am not aware to what extent the remedy may be pushed, nor do I know in what respect very large doses would be deleterious. On these points I should be glad to learn the experience of other practitioners who have largely employed the remedy. I have generally found that the desired effect has resulted after ten-grain doses had been kept up for three or four days, and in cases where the bleeding did not actually cease it was certainly well under control. In several of those painful cases of hemorrhage from fungous growth, the bleeding was much lessened, and the fatal result postponed; in some of my cases I should say that death was due rather to exhaustion and weakening of the general health than to the hemorrhage. I therefore commend this remedy in the cases of hemorrhage to which I have referred, and I prescribe it with confidence, so that its use may be steadily continued until its beneficial action is clearly established.—*Lionel S. Beale, M. B., F. R. S., in The Lancet.*

